# The more the neuroticism, the more the susceptibility to Alzheimer's disease. What inspiration can neuroticism provide?

**DOI:** 10.1002/ibra.12102

**Published:** 2023-05-12

**Authors:** Yifan Yu, Ruitong Yan, Xiaohe Tian

**Affiliations:** ^1^ Department of Radiology and Huaxi MR Research Center (HMRRC), Functional and Molecular lmaging Key Laboratory of Sichuan Province West China Hospital of Sichuan University Chengdu China; ^2^ West China School of Medicine Sichuan University Chengdu Sichuan China; ^3^ Institute for Bioengineering of Catalunya (IBEC) The Barcelona Institute of Science and Technology Barcelona Spain

**Keywords:** cognition and psychiatric disorders, neurodegeneration and repair, neurodegenerative diseases, others

## Abstract

Study of neuroticism can provide important insights. Before the inclusion of neuroticism in the study of Alzheimer's disease (AD), clinical and scientific researchers used relatively fixed models to treat AD, such as prescribing fixed doses of drugs and fixed research strategies. However, taking neuroticism into account affects drug use, the direction of scientific research, and even the mental health of the population, which translates into more immediate economic benefits

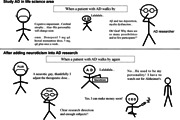

It is often said that “the brain shapes the character”, the idea that “the character influences the brain” is little discussed.

We often believe that “The more you practice, the better you are.” However, this is not entirely true when we look at individuals who are described to be neurotic or prone to distress. It seems that their brains are wired differently: they often have an active mind but unstable control of their emotions, which makes them more prone to negative emotions (such as sadness, depression, and anxiety or other mood disorders).^1^

Exploration of the pathogenesis and treatment strategies of Alzheimer's Disease (AD) shows that there are remarkable similarities between neurotic and AD brains, such as a higher deposition of pathological markers (e.g., amyloid‐beta [Aβ] and tau phosphorylation [Tau]),^2^ more active inflammatory response,^3^ and structural and functional changes^4^ in specific cerebral regions. These similarities led us to hypothesize that neuroticism may be closely related to the onset of AD, and it could provide new insight to overcome some obstacles in traditional AD research.

The enormous burden of AD on patients and their families, the economy, and the society has long been known. Although extensive research on the mechanism of AD has been ongoing for decades, no major breakthrough has been achieved. More importantly, the origins of pathological proteins seen in AD remain unsolved. Understanding the true causes of Alzheimer's requires that at least two major challenges be addressed: (1) the complexity of the disease itself and (2) the “researcher confusion” or “ lack of specificity” caused by the sheer volume of inconclusive research. Thus, study of neuroticism may provide a huge boost towards resolving the second challenge.

Some common themes in current AD research include inflammation, neuronal cell death, pathological protein aggregation, synaptic and neuronal network defects, aberrant proteostasis, cytoskeletal abnormalities, altered energy homeostasis, and DNA and RNA defects.^5^ Despite being closely related to the occurrence of AD, it is often very difficult to translate these findings into clinical applications and drug development due to their complexity. While there are new discoveries related to AD from time to time, a bigger picture is still lacking. Here, we hypothesize that study of neuroticism may provide us with some answers. Neuroticism, as a personality trait, is a psychological state that enables people's tendency to show persistent, stable, and consistent behavior.^6^ Due to the coupling of brain–behavior, micro‐mechanism, and macro‐performance, neuroticism is often accompanied by changes in the aforementioned mechanisms.

It is a well‐known fact that chronic negative experiences cause severe damage to the brain. As a result, many neurological researchers have hypothesized that neurotic brain damage can increase the risk of developing dementia. Currently, these hypotheses have been confirmed to a certain extent through molecular biology, proteomics, genetics, radiology, psychology, and clinical studies. Antonio Terracciano et al.^7^ pooled 20 studies measuring neuroticism using NEO‐personality Inventory‐Revised (NEO‐PI‐R) and found that higher neuroticism scores were associated with more deposition of Aβ and tau, which meant that neurosensitive individuals are more prone to AD‐related pathological changes and may thus be more susceptible to developing AD. In addition, higher neuroticism levels can induce an increase in the density of dopamine receptor 2 (D2)^8^ and the levels of inflammatory cytokine interleukin‐6 (IL‐6),^9^ change the activity of gene loci,^10^ decrease cortical thickness,^11^ and increase levels of cortisol, which may potentially induce damage to the hippocampus.^12^ Qiyong Gong of Huaxi MR Research Center (HMRRC) confirmed the effect of neuroticism on brain neural activity using psychoradiology through a study of resting‐state functional brain imaging.^13^ More importantly, higher levels of neuroticism could increase mortality by inducing an increase in the levels of IL‐6.^14^

In our previous neurological study, we considered the brain as the root of the personality, especially neuroticism, but ignored the effects that the personality could have on the brain. While we focus on the idea that emotional characteristics, such as personality, are brain‐derived, recent research indicates that some emotions can be cardiogenic.^15^ This study shows that when it comes to “traditional brain‐related questions,” we should not just focus on the brain itself but also other elements that may yield more insights. In the field of AD, there is relatively little research on neuroticism, but the results are remarkable. Perhaps it is time for a new approach to the study of AD.

Currently, study of AD in terms of neuroticism has three obvious advantages: (1) it can provide a new perspective of observation: macro to micro. Since neurodegenerative disease like AD can mediate macro performance (e.g., personality traits) through the “molecular‐protein‐structure/function–behavior/personality traits” pathway, the ability of macro performance to influence the micro changes of the brain through the “behavior/personality traits‐structure/function‐protein‐molecular” pathway remains to be explored. In other words, AD can induce changes in the personality, but little is known about whether personalities can affect the onset or development of AD. (2) The evaluation dimension can be reduced and this can enable specific focus on the problem. Neuroticism is a comprehensive indicator, which is related not only to pathological markers but also some hormones and submicroscopic molecules, such as glucocorticoids^16^ (which may cause hippocampal neuron damage in AD patients), microtubule‐associated tau protein (MAPT), and a common posttranscriptional messenger RNA modification N^6^‐methyladenosine quantitative trait loci (m^6^A QTLs).^17^ It is worth mentioning that the m^6^A QTLs can cause similar responses in different organs and regulate the overall reactivity of the body by controlling the transcription expression. High expression of m^6^A QTLs is associated with neuroticism, depression, and anxiety in the brain, asthma in the lung, and coronary artery disease in the heart. Thus, we can speculate that neuroticism will also have similar effects on the body through a certain pathway, like m^6^A QTLs, to improve the body's reactivity, so as to make it easier to reach the threshold for the occurrence of AD pathological lesions. (3) Personalized interventions can be designed. The intervention for neuroticism is different from a conventional lifestyle intervention, as the former is specific and the latter is general. Individuals with high levels of neuroticism have higher resting activity in the left middle temporal gyrus, left striatum, and right hippocampus and lower activity in the left superior temporal gyrus and right striatum.^13^ We are aware that different people with different traits will have different performances in neural active areas and neural activity; thus, the sensitivity to drugs or other therapeutic methods is different. Due to the homogeneous nature of neuroticism, it is considered “derivable,” which can help provide more personalized guidance for intervention strategies in AD like adjustment of the therapeutic dose (drug dosage or physical therapy). These advantages may provide some inspirations for the study of AD. The inclusion of neuroticism in the study of AD may have implications in scientific research, clinical studies, and medicine, and may even benefit the general public (Figure 1).

**Figure 1:**
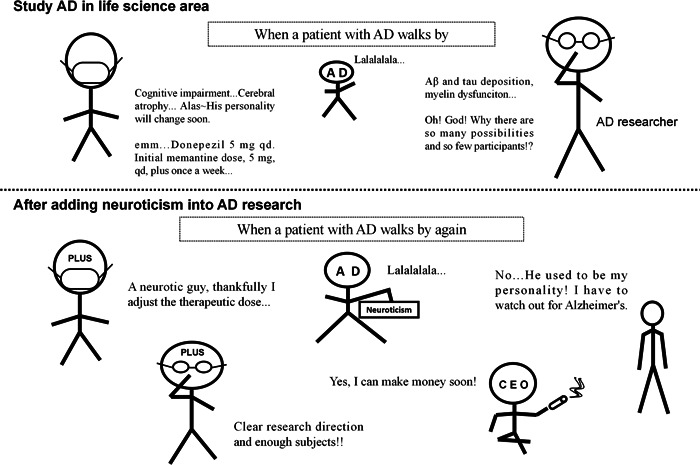
Insights that neuroticism can provide. Before the inclusion of neuroticism in the study of Alzheimer's disease (AD), clinical and scientific researchers used relatively fixed models to treat AD, such as prescribing fixed doses of drugs and fixed research strategies. However, taking neuroticism into account affects drug use, the direction of scientific research, and even the mental health of the population, which translates into more immediate economic benefits.

Yet, a number of obstacles remain to be overcome in the study of neuroticism (Figure 2): (1) Evaluation errors caused by confounding factors. For example, if a person is depressed, it may enhance his or her perception of vulnerability and thus result in an increase in the neuroticism score on the scale. Therefore, how many confounding factors should we consider in neuroticism research? Do these confounding factors strengthen or weaken the results? What measures should be taken to minimize the impact of confounding factors? (2) Subjectivity. Variations in diagnostic standards, clinical proficiency, and personal subjective emotional experience are common sources of heterogeneity in neuroticism test results. Ways to obtain more objective evidence or more uniform subjective evaluation results are the key to enabling generalization of the results of the study of neuroticism in the future. (3) Specificity. Neuroticism is a macroscopic manifestation of various brain activities, including the speed and efficiency of signal transmission in the brain, the level of inflammation, the degree of immune activation, the accumulation of pathological substances, and the willingness to improve one's mental health. Meanwhile, personality traits such as neuroticism affect not just the nervous system but also the circulatory and other systems. Therefore, the extent to which neuroticism might be related to AD remains to be proven. (4) Lack of intervention strategies for neuroticism. Even though psychotherapy has been found to significantly improve outcomes for many diseases and psychological counseling has been found to have the potential to modify neuroticism by changing the way clients perceive things, it is unclear how effective it can be. In addition to the four points mentioned above, perhaps the biggest dilemma of advancing AD research by the study of neuroticism is that we need to determine the role that neuroticism plays in the different changes that occur in AD patients. How can we study this? Does neuroticism affect AD through immune mechanisms, inflammation, neuroendocrinology, changes in cellular electrophysiology, or in other ways? Also, we need to determine what kind of experimental paradigms we can develop to study neuroticism. These questions are fundamental to whether we can actually consider the study of neuroticism as a critical field in the research of AD.

**Figure 2:**
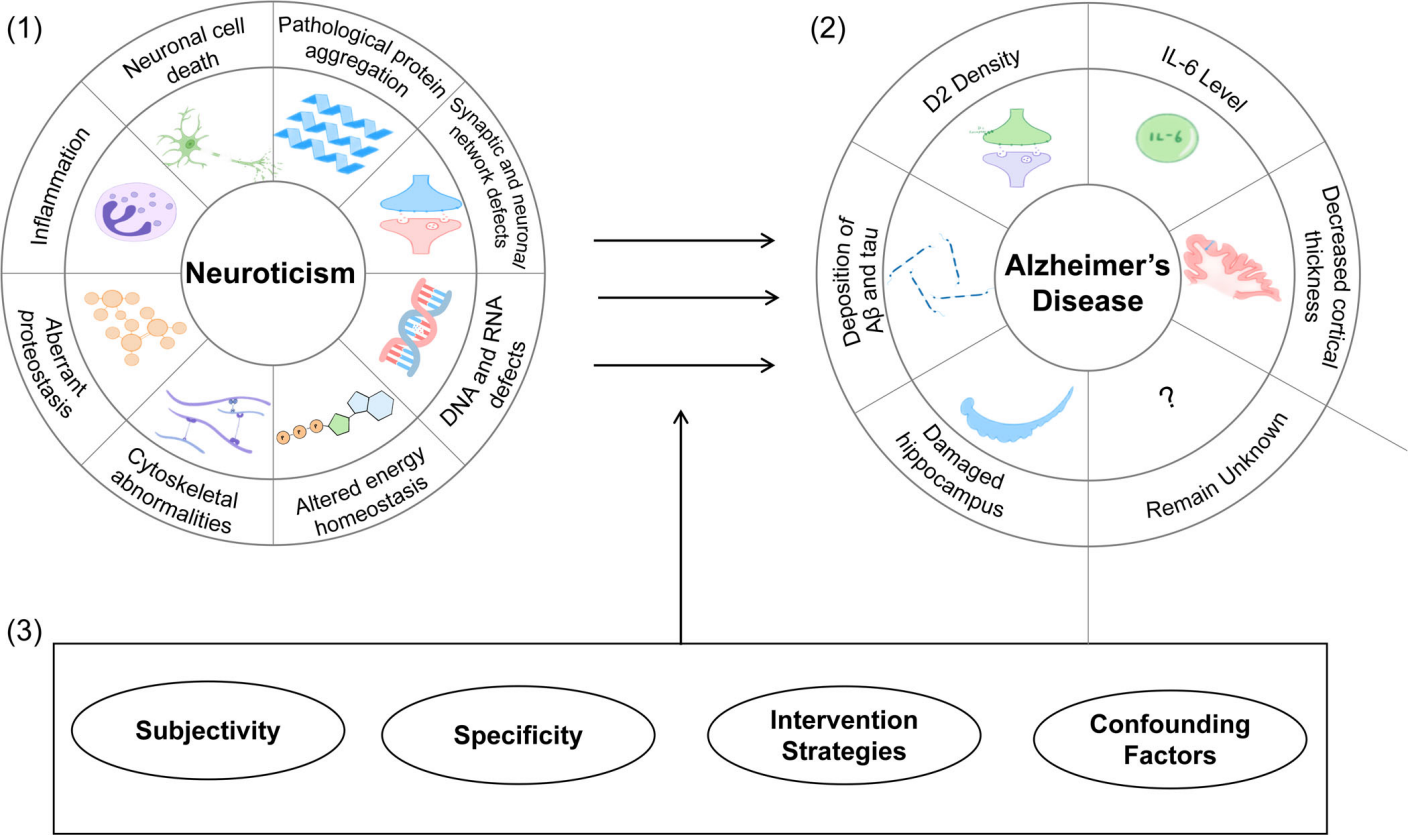
Summary of neuroticism and Alzheimer's disease (AD). (1) Potential mechanisms by which neuroticism acts on AD (based on the existing research field of AD): inflammation, neuronal cell death, pathological protein aggregation, synaptic and neuronal network defects, aberrant proteostasis, cytoskeletal abnormalities, altered energy homeostasis, and DNA and RNA defects. (2) Proven effects of neuroticism on AD: damaged hippocampus, deposition of Aβ and tau, D2 density, IL‐6 level, decreased cortical thickness, and other potential mechanisms that remain unknown. (3) Limitations of exploring AD by the study of neuroticism: Subjectivity, specificity, intervention strategies, and confounding factors. The combination of these three is intended to show that neuroticism can affect the molecular, pathological, and other manifestations of AD through specific mechanisms, thus establishing a “Neuroticism–AD” pathway. However, the successful establishment of this pathway depends on whether some limitations of neuroticism can be resolved. [Color figure can be viewed at wileyonlinelibrary.com]

Finally, for those with high levels of neuroticism, the following question may arise: “Are there any other ‘rescues’ in the absence of a specific effective intervention strategy for neuroticism?” Some studies have shown that neuroticism develops primarily early in life and is relatively stable in adulthood.^18^ Indeed, besides neuroticism, three personality traits— conscientiousness, agreeableness, and openness—were negatively correlated with the deposition of Aβ and tau.^2^ This suggested that even though some individuals may have high levels of neuroticism in general, we can take many strategies to make ourselves responsible, outgoing, and comfortable to counterac the extra risk of AD associated with neuroticism.

Clinical experience has long taught us that the nervous system and mental health are inextricably linked, but how the two interact remains unknown. The main reason for this lack of clarity is that we cannot even find a suitable research topic that can act as a bridge for us to study both. Since *Neurology's* “At my wits' end” comment on Neuroticism and AD 20 years ago,^19^ increasingly more studies have found a relationship between neuroticism and AD. While researchers are trying to further advance the diagnosis and treatment of AD by studying neuroticism, they are also hoping to find a link between neurosis and spirituality through the study of neurosis and personality. Now, we know that neuroticism is strongly associated with the occurrence of pathological manifestations, increased risk, and progression of symptoms of AD. If the correlation between AD and neuroticism can be further clarified, it will not only be beneficial for the treatment of neurodegenerative diseases such as AD but will also be a bridge between neurology and psychiatry.

## AUTHOR CONTRIBUTIONS

**Yifan Yu**: Idea conceptualization, article collection, and curation, original draft writing. **Yifan Yu** and **Ruitong Yan**: Manuscript drafting and visualization. **Xiaohe Tian**: Review, editing, and supervision.

## CONFLICT OF INTEREST STATEMENT

Prof. Xiaohe Tian is the associated editor of Ibrain. He has fully revealed these interests, and has worked out a plan for approval to manage any potential conflicts caused by the participation. Other authors have no conflict of interest to disclose.

## ETHICS STATEMENT

This article does not contain any studies with human or animal subjects. Ethical approval is not applicable for this article.

## Data Availability

The data are available.
